# Genetic variant rs*10251977 (G*>*A)* in EGFR-AS1 modulates the expression of EGFR isoforms A and D

**DOI:** 10.1038/s41598-021-88161-3

**Published:** 2021-04-22

**Authors:** Shankar Dhamodharan, Mathew Maria Rose, Sundaram Reddy Chakkarappan, Karuppiah Vijayamuthuramalingam Umadharshini, Ramalingam Arulmurugan, Shanmugam Subbiah, Ituro Inoue, Arasambattu Kannan Munirajan

**Affiliations:** 1 Department of Genetics, Dr. ALM PG Institute of Basic Medical Sciences, University of Madras, Taramani Campus, Chennai, 600 113 India; 2 Center for Oncology, Royapettah Government Hospital & Kilpauk Medical College, Royapettah, Chennai, 600 014 India; 3 Division of Human Genetics, National Institute of Genetics, Mishima, 411-8540 Japan; 4 Department of Health Research, Multi Disciplinary Research Unit (DHR-MRU), Dr. ALM PG Institute of Basic Medical Sciences, University of Madras, Taramani Campus, Chennai, 600 113 India

**Keywords:** Cancer, Genetics, Molecular biology, Oncology

## Abstract

Tyrosine kinase inhibitor is an effective chemo-therapeutic drug against tumors with deregulated EGFR pathway. Recently, a genetic variant *rs10251977* (G>A) in exon 20 of *EGFR* reported to act as a prognostic marker for HNSCC. Genotyping of this polymorphism in oral cancer patients showed a similar frequency in cases and controls. EGFR-AS1 expressed significantly high level in tumors and EGFR-A isoform expression showed significant positive correlation (r = 0.6464, p < 0.0001) with reference to EGFR-AS1 expression levels, consistent with larger TCGA HNSCC tumor dataset. Our bioinformatic analysis showed enrichment of alternative splicing marks H3K36me3 and presence of intronic polyA sites spanning around exon 15a and 15b of EGFR facilitates skipping of exon 15b, thereby promoting the splicing of EGFR-A isoform. In addition, high level expression of PTBP1 and its binding site in EGFR and EGFR-AS1 enhances the expression of EGFR-A isoform (r = 0.7404, p < 0.0001) suggesting that EGFR-AS1 expression modulates the EGFR-A and D isoforms through alternative splicing. In addition, this polymorphism creates a binding site for miR-891b in EGFR-AS1 and may negatively regulate the EGFR-A. Collectively, our results suggested the presence of genetic variant in EGFR-AS1 modulates the expression of EGFR-D and A isoforms.

## Introduction

Oral squamous cell carcinoma (OSCC) is one of the prevalent cancers worldwide. According to the GLOBOCAN 2018 report from India, cancer of lip, oral cavity is the top most cancer in men and fourth most in women^1^ and often diagnosed in advanced stages, making it difficult for the therapeutic management. Tobacco chewing/smoking, alcohol consumption and infection with human papilloma virus (HPV) 16/18 are the major risk factors of OSCC^2^. In India, tobacco chewing with betel quid, slaked lime, and areca nut combined with smoking or drinking significantly increases the risk. Despite the advances in diagnosis and treatment the mortality rate of oral cancer patients has not markedly improved over the past three decades and the 5-year survival rate remains less than 50%. Even with the advances in drug discovery and treatment against cancer, chemoresistance and tumor recurrence remains an obstacle for development of effective therapeutic management in the patients with OSCC^3,4^.

The etiology (58–90%) of HNSCC is attributed with the aberrant activity of epidermal growth factor receptor (EGFR). It belongs to the family of receptor tyrosine kinases ErbB and plays an important role in cell cycle regulation, proliferation, cell migration and other physiological processes^5^. Overexpression of EGFR is observed in the early stages of the oral tumorigenesis and linked with the advanced stages of the tumor, thus it could serve as the effective drug target^6^. Though various evidences proved EGFR signalling is associated with progression of HNSCC, the effective treatment outcome is not achieved upon targeting EGFR with monoclonal antibodies and/or TKIs^7^. Therefore, it is essential to identify robust biomarkers for the success of targeted therapy.

Alternative splicing is a common mechanism employed by eukaryotic cells to create diversified proteomics profile from single gene^8,9^. This mechanism is common in receptor kinase family genes. EGFR generates a functionally important four splice variants through alternative splicing where 10.5 kb and 5.8 kb belongs to variant 1 class encoding 170 kDa protein called isoform A. This variant will be generated by skipping of two exons 15a and 15b in *EGFR*, whereas 1.8 kb, 2.4 kb and 3.0 kb are three other isoforms formed from a read-through of a exon–intron boundary and incorporation of alternate exons 15a and 15b, which encodes 60, 80 and 110 kDa proteins known as isoform B, C and D, respectively^10,11^.

Currently, long non-coding RNAs (lncRNAs), a class of heterogeneous RNA molecules (> 200 nt) with no protein-coding potential, has been identified as the prognostic marker in different cancer types. The lncRNAs were aberrantly expressed in various cancers and play regulatory roles in several cellular pathways promoting proliferation, stem cell pluripotency, cellular reprogramming, cellular transformation, and tumorigenesis^3^. A recent study established the prognostic potential of EGFR-AS1 in tyrosine kinase inhibitors (TKIs) treatment and knockdown of EGFR-AS1 induced regression of squamous cell carcinoma of head and neck^6^.

Antisense non-coding transcript of *EGFR* locus (EGFR-AS1), a 2.8 kb transcript and was shown to promote the stability of its *cis* partner EGFR-A isoform^12^. However, the role of EGFR-AS1 in maintaining the stability of EGFR-A isoform is yet to be studied in detail. This study is focussed on understanding the molecular consequence of a genetic variant *rs10251977* (c.2361G>A) in EGFR-AS1 that creates a binding site for miR-891b and modulates the stability of the EGFR-AS1. Additionally, we explored the role of EGFR-AS1 in the alternative splicing of EGFR A/D isoforms.

## Results

### Genotype frequency of genetic variant *rs10251977* in oral cancer patients of South Indian origin

We genotyped the genetic variant *rs10251977* (c.2361G>A) in exon 20 of *EGFR* in 180 oral cancer patients with age and sex matched 184 cancer-free controls. The demographic details of both cases and controls were presented in Table 1. Oral cancer patients and control subjects’ genotype frequencies were in agreement with Hardy–Weinberg equilibrium with p values 0.79 and 0.35, respectively (Table 2). The genotype frequencies were found to be 40% (72/180) of GG, 47.2% (85/180) of GA and 12.8% (23/180) of AA in oral cancer patients, 36.4% (67/184) of GG, 50.5% (93/184) of GA and 13.1% (24/184) of AA in control subjects and no significant difference was found between cases and controls. A similar result was observed in dominant model (GA + AA vs GG), recessive model (GG + GA vs AA) and allelic model (G vs A) with an OR of 0.86 (p = 0.55), 0.98 (p = 0.93) & 0.92 (p = 0.64), respectively.

**Table 1:** Demographic and clinical data of oral cancer patients and healthy controls.

Clinical parameters	Oral cancer patients (n = 180)	Controls (n = 184)
Age in years, Mean ± SD	53.1 ± 11.0 (26 – 80)	50.4 ± 9.3 (25 – 82)
Sex (Male / Female)	124/56	125/59
**Risk habits N (%)**		
Smoking	96 (53.3)	9 (4.9)
Chewing	75 (41.6)	–
Alcoholics	46 (25.5)	4 (2.2)
Smoking + chewing	25 (13.8)	–
Smoking + alcoholics	16 (8.8)	55 (29.9)
Smoking + chewing + alcoholics	19 (10.5)	–
No risk habits	42 (23.3)	116 (63)
**Histological differentiation N (%)**		
Poor	26 (14.4)	–
Moderate	77 (42.8)	–
Well	77 (42.8)	–
**Tumor stage N (%)**		
≤ T2	34 (18.9)	–
>T2	146 (81.1)	–
**Nodal invasion N (%)**		
Positive	150 (83.3)	–
Negative	30 (16.7)	–

**Table 2:** Genotype, Allele frequency in South Indian oral cancer patients and controls.

*rs10251977*; c.2361G>A		Oral cancer N (%)	Control N (%)	Odds ratio	95% CI	p-value
Genotype	GG	72 (40)	67 (36.4)	1 (Ref)		
	GA	85 (47.2)	93 (50.5)	1.17	0.75–1.83	0.47
	AA	23 (12.8)	24 (13.1)	1.12	0.57–2.17	0.73
Dominant model	GA + AA	108 (60)	117 (63.6)	0.86	0.56–1.31	0.55
Recessive model	GG + GA	157 (87.2)	160 (87)	0.98	0.53–1.80	0.93
Allelic model	G	229 (63.6)	227 (61.7)	1 (Ref)		
	A	131 (36.4))	141 (38.3)	0.92	0.68–1.24	0.64
HWE χ^2^		0.072	0.88			
HWE p-value		0.79	0.35			

### EGFR-AS1 is overexpressed in OSCCs

**Figure 1:**
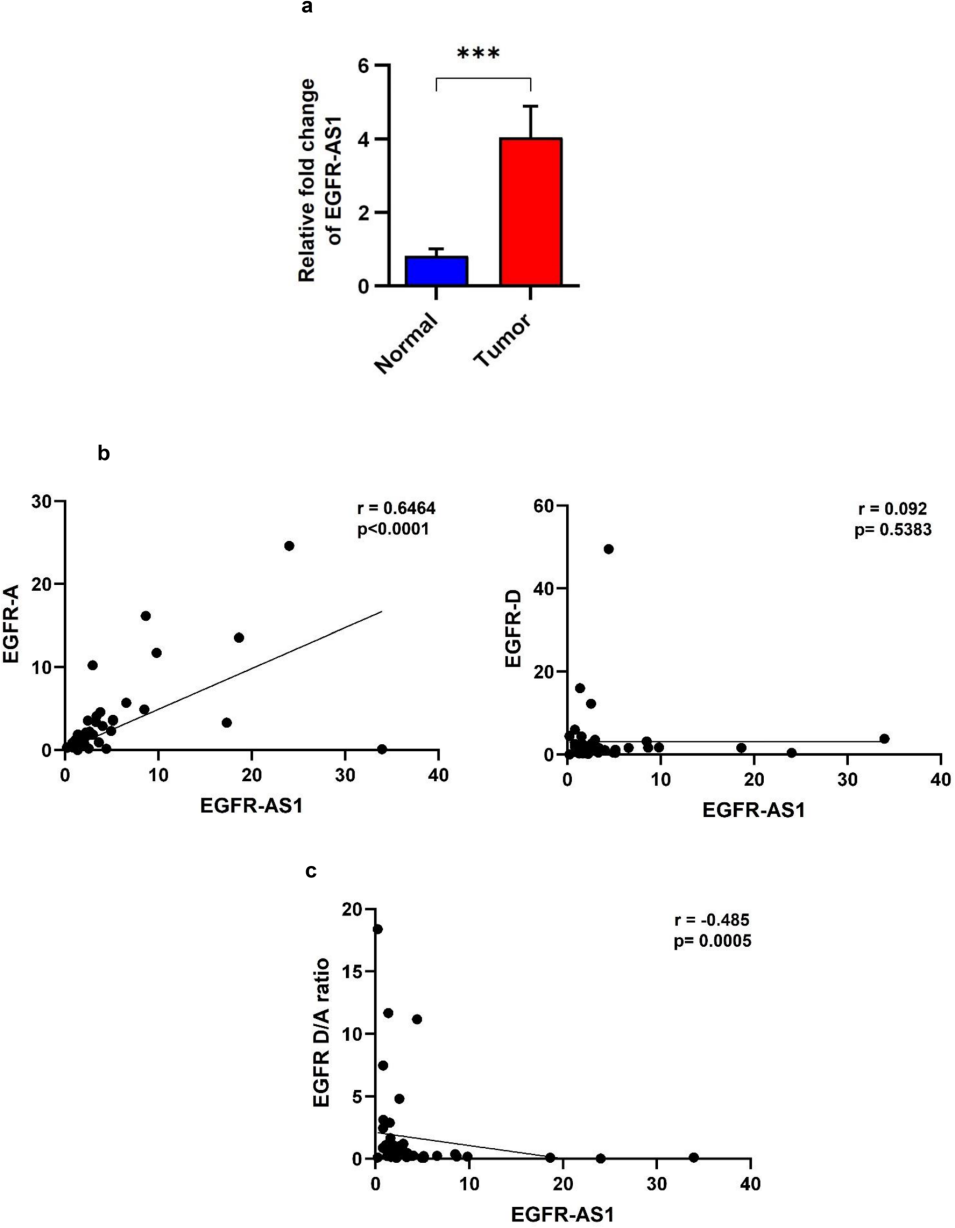
Role of EGFR-AS1 in maintaining the level of EGFR A and D isoforms in OSCC. (**a**) Relative expression level of lncRNA EGFR-AS1 in oral tumors compared with normal tissues. (**b**) Scatter plot showing the correlation analysis of EGFR-A and D isoforms expression in relation to EGFR-AS1 expression. (**c**) Scatter plot showing the EGFR D/A ratio in relation to EGFR-AS1 expression. (Statistical analysis was done by, two tailed Student’s t-test, Spearman rank correlation analysis, p-value *** for < 0.001).

The EGFR-AS1 expression was analyzed in 48 oral tumor samples and 8 independent normal tissues by RT-qPCR (Table 3). A significant upregulation of EGFR-AS1 was observed in tumor tissues compared to that of normal tissues (p < 0.001) (Fig. 1a). When the expression levels of EGFR-AS1 was correlated with clinic-pathological characteristics of the tumors, there is an increased level of EGFR-AS1 expression without any statistically significant association (Supplementary Fig. S1a). Interestingly, in tumors with GA and AA (N = 25) genotypes, we observed high level of EGFR-AS1 expression albeit with no statistical significance (p = 0.653), and this could be due to the small sample size (Supplementary Fig. S1b).

**Table 3:** Relationship between EGFR-AS1 expression and clinicopathological characteristics in oral cancer patients.

Clinical characteristics	EGFR-AS1 expression level		p-value^#^	OR	95% CI
	Low (N = 24)	High (N = 24)			
**Age (years)**					
≤ 51	11	14	0.5634	0.6044	0.1929–1.893
>51	13	10			
**Sex**					
Female	6	5	0.999	1.267	0.3281–4.891
Male	18	19			
**Tobacco habits**					
Smoking					
Yes	17	14	0.546	1.735	0.5238–5.745
No	7	10			
**Chewing**					
Yes	10	10	0.7697	1	0.3173–3.152
No	14	14			
**Histological differentiation**					
Poor and moderate	17	14	0.546	1.735	0.5238–5.745
Well	7	10			
**Tumor stage**					
≤ T2	3	8	0.831	0.8571	0.1736–4.233
>T2	21	16			
**Nodal invasion**					
Positive	22	20	0.1905	2.2	0.3627–13.34
Negative	2	4			

^#^Fisher’s exact test with two tailed P value.

### EGFR-AS1 expression level correlates with the EGFR A and D isoforms expression

To know the functional relationship of EGFR-AS1 expression with EGFR isoforms, we analysed the relative expression levels of EGFR isoforms A and D and D/A ratio with reference to the expression level of EGFR-AS1. The tumor samples were stratified into two groups as EGFR-AS1 high and low expression groups based on the median values of EGFR-AS1 expression (Table 3). Tumors with expression level above median value is considered as high expression group and those with fold change below median value as low expression group. We observed a statistically significant difference in the expression level of EGFR-A isoform with reference to EGFR-AS1 level (r = 0.6464, p < 0.0001) and no significant difference was found for EGFR-D isoform (r = 0.092, p = 0.5383) (Fig. 1b). However, there was significant negative correlation between the EGFR isoform A and D levels in relation to EGFR-AS1 level (r = − 0.485, p = 0.0005) (Fig. 1c) and this could be due to the modulation of RNA splicing. To address this, we carried out in-silico analysis to identify the EGFR-AS1/EGFR binding partners that has functional association with alternative splicing.

**Figure 2:**
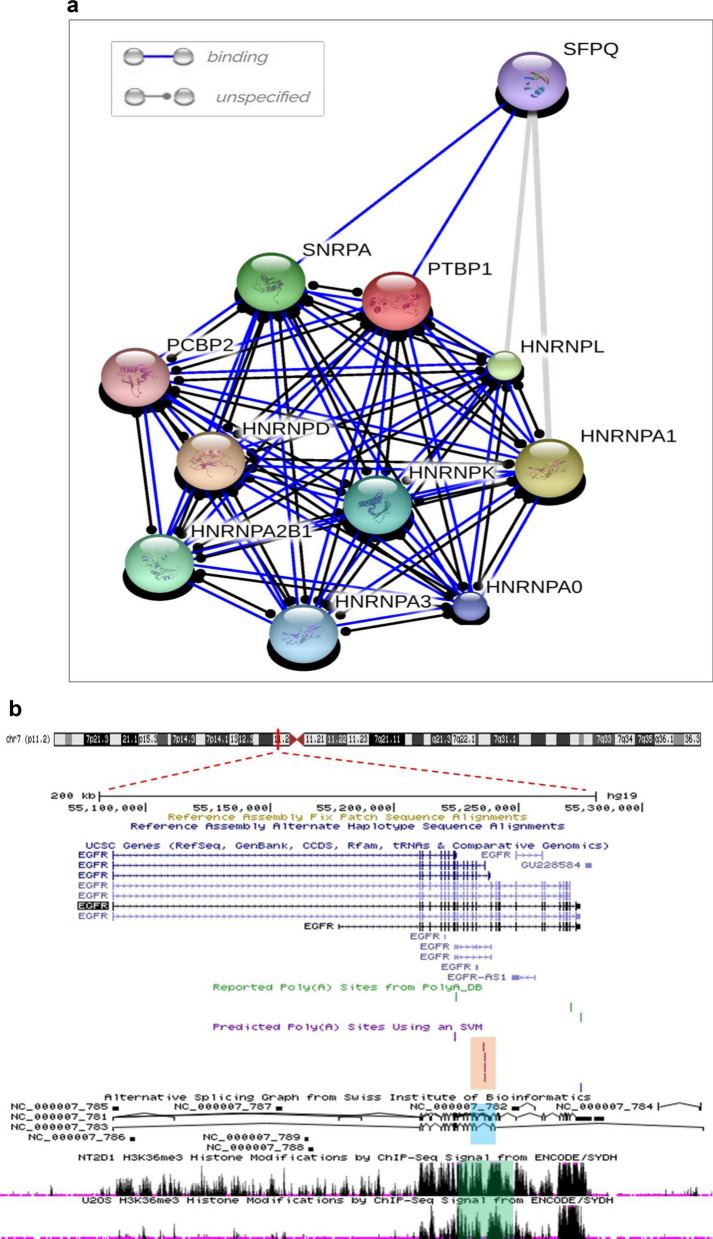

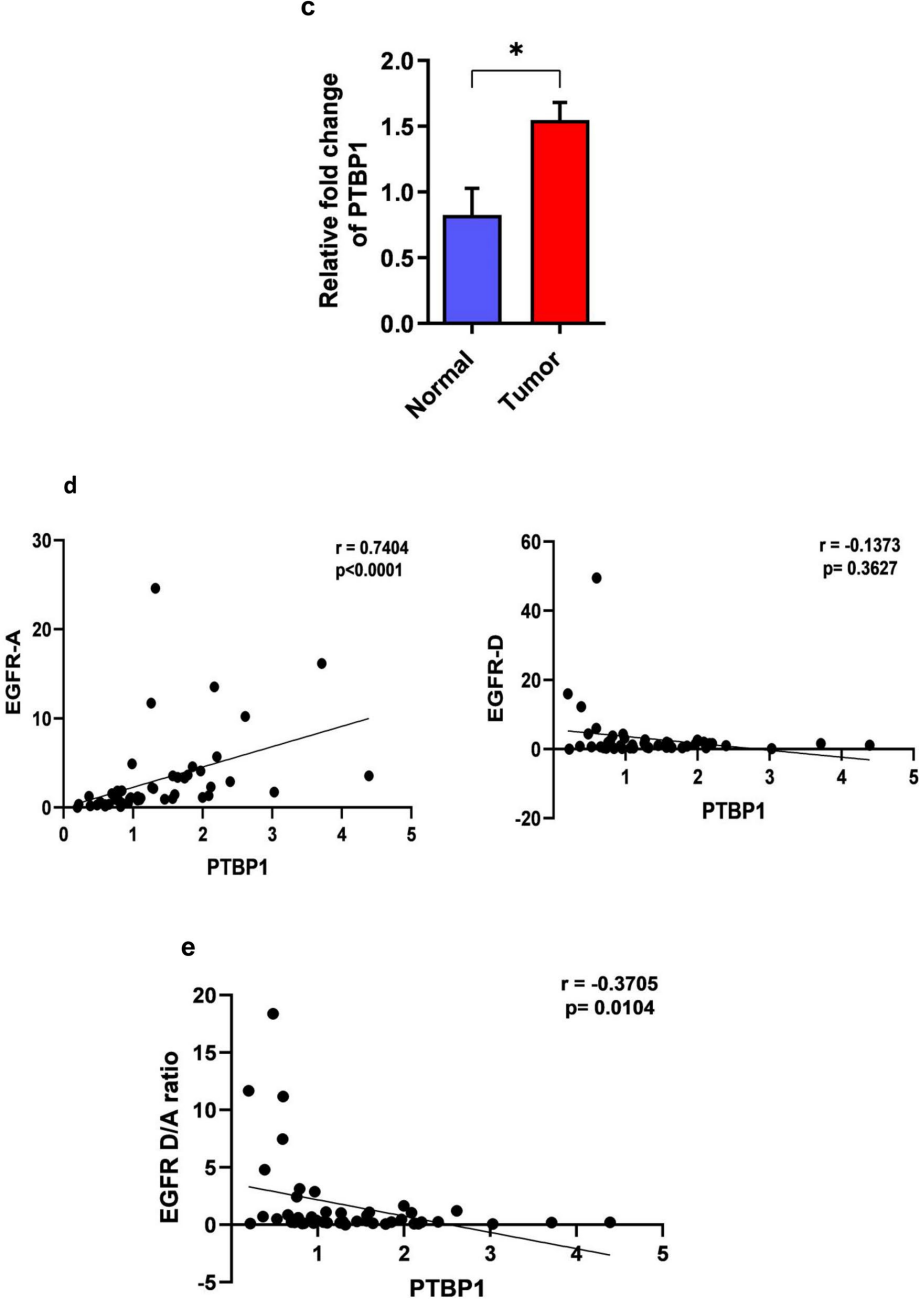
In-silico characterization of EGFR-AS1 role in alternative splicing of EGFR. (**a**) STITCH analysis showing the binding partners of PTBP1. (**b**) UCSC genome browser data shows the H3K36me3 marks (green box) and polyA sites (orange box) prevalence between exon 15 and 16 skipped region (blue box) of EGFR. (**c**) Relative expression level of PTBP1 in oral tumors compared with normal tissues. (**d**) Scatter plot showing the correlation analysis of EGFR-A and D isoforms expression in relation to PTBP1 expression. (**e**) Scatter plot showing the EGFR D/A ratio in relation to PTBP1 expression. (Statistical analysis was done by, two tailed Student’s t-test, Spearman rank correlation analysis, p-value * for < 0.01).

First, we predicted the binding partners of EGFR-AS1 by using online database lncRNAtor^13^. EGFR-AS1 showed significant mechanistic association with three RNA binding proteins FIP1, hnRNPU, and PTBP1, of which PTBP1 showed a significant association (p = 8.43E^−26^) (Supplementary Fig. S2a). To evaluate the binding of this alternative splicing factors in *EGFR* locus we used RBPmap online tool^14^ to predict the binding motif of the RNA binding proteins in both EGFR-AS1 and region between exon 15 and 16 of *EGFR* gene and identified several consensus motif of PTBP1 (Supplementary Tables S1 and S2), suggesting the PTBP1 role in alternative splicing of EGFR-A isoform. To further support this hypothesis, we checked the binding partners for PTBP1 using STITCH online tool, and found that most of its interacting partners HNRNPK, HNRNPU, HNRNPA1, HNRNPA3, HNRNPD, HNRNPL and SNRPA were known to play a role in alternative splicing (Fig. 2a).

Previous studies have shown that H3K36me3 plays an important role in exon skipping by recruiting alternative splicing factors^15^. We checked whether the H3K36me3 marks are present in EGFR region by using UCSC genome browser. Interestingly, we found that enhanced H3K36me3 marks were present around the skipped region spanning the exon 15a and b region (Fig. 2b), and to our surprise they also carried a few polyA sites spanning around region exon 15a leading to the skipping of exon 15b resulting in the reduction of EGFR-D isoform transcript. In support to our prediction, we observed significant upregulation of PTBP1 expression in tumor samples compared to normal (p = 0.0467) (Fig. 2c) and it showed significant positive correlation with EGFR-A isoform (r = 0.7404, p < 0.0001) (Fig. 2d). Though we did not obtain significant negative correlation with EGFR-D isoform (r = − 0.1373, p = 0.3627) (Fig. 2d), we found significant negative correlation between PTBP1 and EGFR-D/A ratio (r = − 0.3705, p = 0.0104) (Fig. 2e). Consistent with our findings, TCGA data analysis (Supplementary Fig. S2b,c) also showed elevated PTBP1 expression and its association with EGFR-A isoform. Thus, EGFR-AS1 may act as a scaffold to recruit major alternative splicing factors in association with PTBP1 to bring exon skipping and promoting EGFR-A expression.

### EGFR variant *rs10251977* creates miR-891b binding site in EGFR-AS1

Apart from acting as scaffold, lncRNAs may also act as miRNA sponges in the cytoplasm. Tan et al., reported that the presence of minor allele in EGFR-AS1 decreased its steady state level^7^ and this could be due to the miRNA sponging mediated degradation of lncRNA. We used a web-based tool called lncRNASNP2 to determine the functional impact of minor allele in EGFR-AS1.

**Figure 3:**
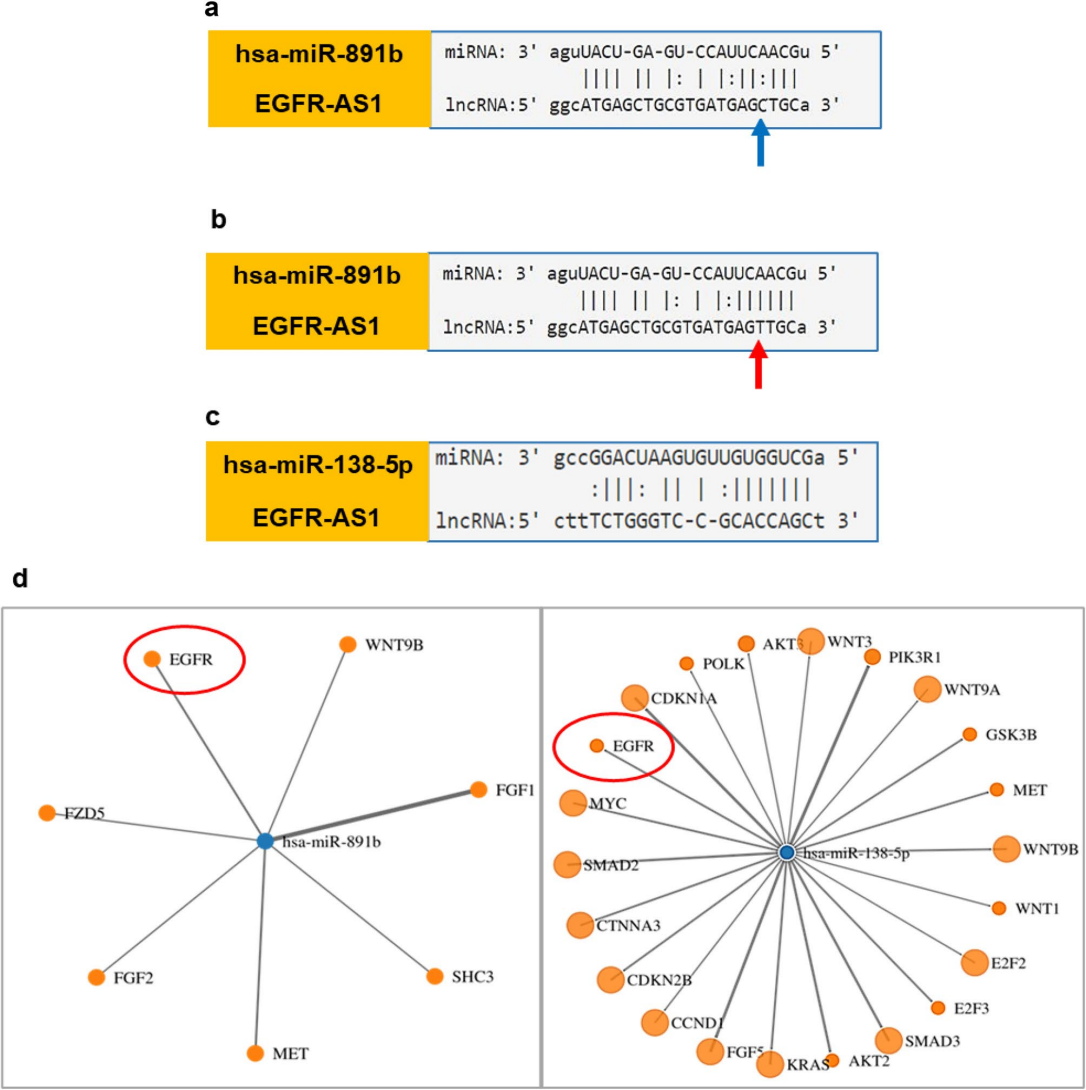
*rs10251977* minor allele’s effect on EGFR-AS1. (**a**) Absence of minor allele in EGFR-AS1 (Blue arrow). (**b**) Prediction of effect of minor allele in EGFR-AS1 generating binding site for miR-891b using lncRNASNP2 (Red arrow indicating presence of variant allele). (**c**) Figure showing the binding site for another miRNA, miR-138-5p in EGFR-AS1. (**d**) miRWalk database showing the targets of both miR-891b and miR-138-5p (EGFR encircled in both miRNAs).

The minor allele generates a new binding site for miR-891b (Fig. 3a,b). We chose this miRNA and another miRNA, miR-138-5p (Fig. 3c) which targets EGFR and remains unaffected by the presence of either major or minor allele for expression study (Supplementary Fig. S2d). Both the miRNAs targets EGFR-A isoform, as confirmed by TargetScan and miRwalk tools (Fig. 3d). Gene Set Enrichment Analysis (GSEA) for miR-891b and miR-138-5p revealed that they are involved in pathways related to cancer and MAPK signaling pathway, respectively (Supplementary Tables S3 and S4).

### Correlation of EGFR-AS1 level with the miRNA level

**Figure 4:**
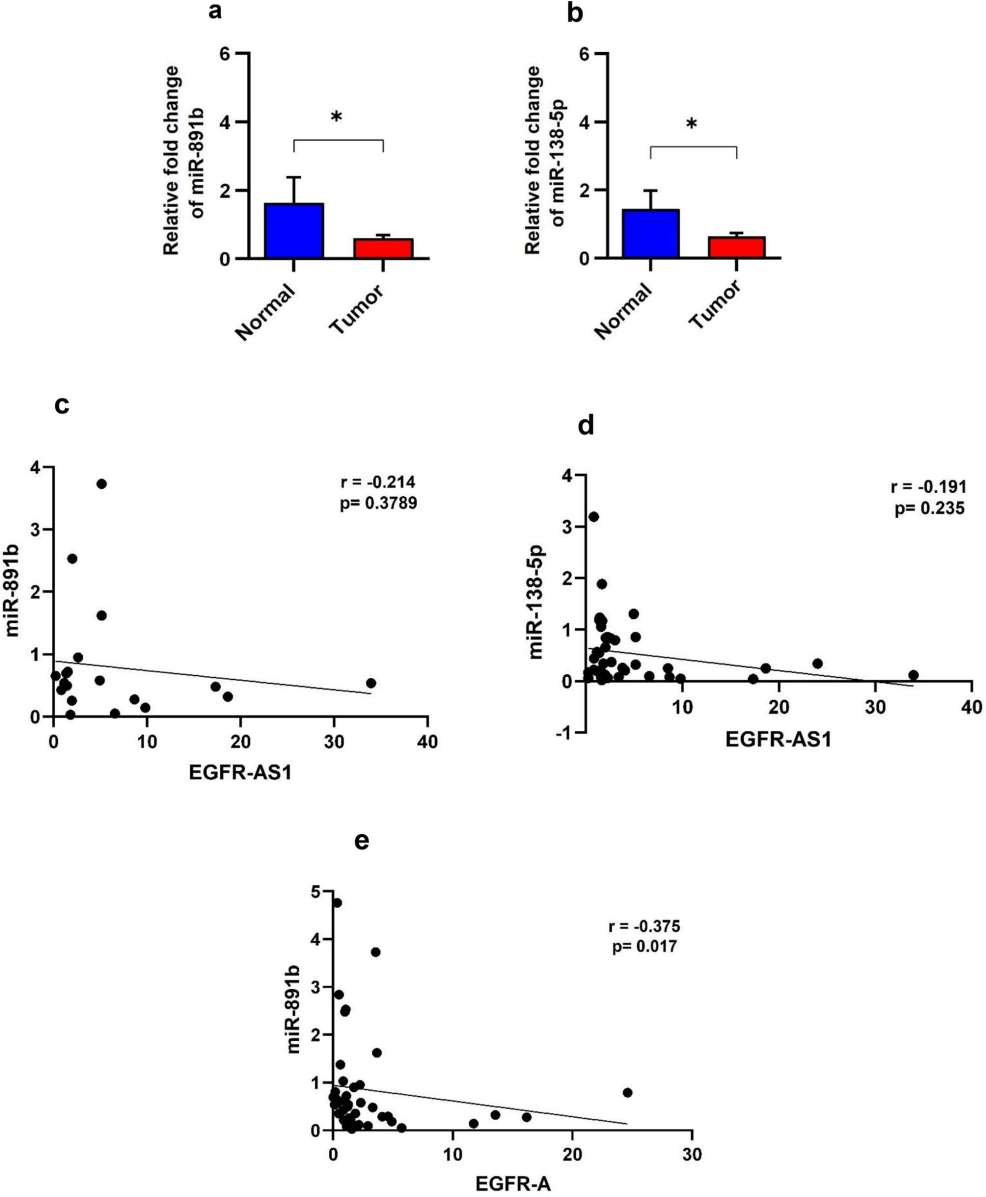
miR-891b sponging by EGFR-AS1 promoting the expression of EGFR-A isoform. (**a**) Graph showing the relative fold change of miR-891b. (**b**) Graph showing the relative fold change of miR-138-5p. (**c**) Scatter plot showing the correlation analysis of EGFR-AS1 in relation to miR-891b level. (**d**) Scatter plot showing the correlation analysis of EGFR-AS1 in relation to miR-138-5p level. (**e**) Scatter plot showing the correlation analysis of EGFR-AS1 in relation to miR-891b. (Statistical significance represented as * for p-value < 0.01, two tailed Student’s t-test and Spearman rank correlation analysis).

The expression levels of both miR-891b and miR-138-5p, were analyzed in 48 oral tumor tissues and 8 normal tissues and were found to be significantly downregulated in tumor tissues compared to that of normal tissues with a p-value of 0.03 and 0.047, respectively (Fig. 4a,b). Both miRNAs, miR-891b and miR-138-5p showed a trend of negative correlation with EGFR-AS1 levels (r = − 0.214, p = 0.3789 and r = − 0.191, p = 0.235, respectively) (Fig. 4c,d). The EGFR D/A isoforms ratio showed a trend of negative correlation with miRNAs level (Supplementary Fig. S3a,b). When correlated with the expression level of EGFR-A, the tumors with an increased level of miR-891b had significantly low level of EGFR-A (r = − 0.375, p = 0.017) (Fig. 4e) suggesting the possibility of ceRNA network operating in these tumors. Our results suggest that the presence of minor allele generates the binding site for miR-891b thereby switching the mechanism of EGFR-AS1 from a scaffold to miRNA sponge modulating the expression level of EGFR-A isoform.

### Correlation of EGFR A and D isoform, EGFR-AS1 and miR-891b expression levels in HNSCC TCGA dataset

**Figure 5:**
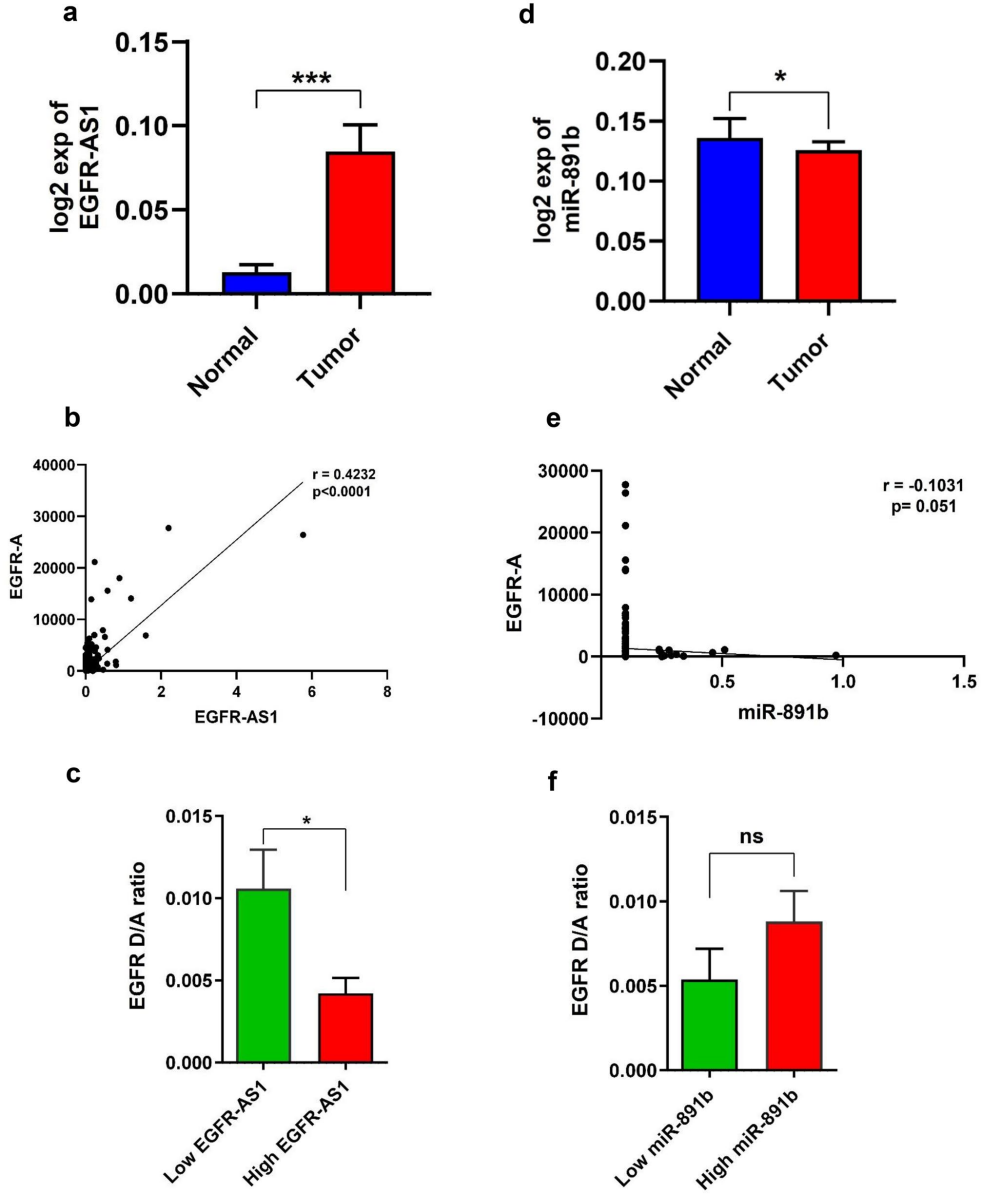
Expression analysis of EGFR A/D, EGFR-AS1 and miR-891b in HNSCC TCGA dataset. (**a**) Graph showing the relative fold change of EGFR-AS1. (**b**) Scatter plot showing the correlation analysis of EGFR-A isoform expression in relation to EGFR-AS1 expression. (**c**) Graph showing the EGFR D/A ratio in relation to low and high EGFR-AS1 expression. (**d**) Graph showing the relative fold change of miR-891b. (**e**) Scatter plot showing the correlation analysis of EGFR-A in relation to miR-891b. (**f**) Graph showing the EGFR D/A ratio in relation to low and high miR-891b expression (Statistical significance represented as * for p-value < 0.01, *** for < 0.001 ns-non significant, two tailed Student’s t-test and Spearman rank correlation analysis).

To confirm the above findings in larger samples, we used HNSCC TCGA datasets (Tumor N = 520, Normal N = 44), derived from TSVdb^16^, TANRIC^17^ and OMCD^18^ to obtain the expression levels of EGFR A and D isoforms, EGFR-AS1 and miR-891b respectively. The splice variants EGFR A and D is given in Supplementary Fig. S4. Tumors with expression level above median value is considered as high expression group and the expression level below median value as low expression group. HNSCC TCGA datasets also showed a significant upregulation of EGFR-AS1 in tumors compared to the normal tissue (p = 0.0008) (Fig. 5a) in consistent with our findings. In addition, we found a significant positive correlation between EGFR-AS1 and EGFR-A expression (r = 0.4232, p < 0.0001) (Fig. 5b). EGFR D/A ratio was significantly reduced in EGFR-AS1 high expressing tumors compared to the low expression group (p = 0.014) (Fig. 5c). With reference to miR-891b expression in TCGA datasets, we observed a significant downregulation in tumor tissues compared to the normal tissue (p = 0.042) (Fig. 5d) confirming the results of this study. However, there is no strong correlation between miR-891b level and EGFR-A level (r = − 0.1031, p = 0.051) (Fig. 5e), but there was trend towards the negative regulation of EGFR D/A ratio in miR-891b high expressing tumors (p = 0.8828) (Fig. 5f). Collectively, the TCGA data strongly supports the role of EGFR-AS1 in controlling EGFR isoforms.

## Discussion

*EGFR* is the most frequently mutated and deregulated gene in multiple cancers, especially cancers of epithelial origin in humans making tyrosine kinase inhibitors as an effective targeted therapy in those cancers^19–21^. Various studies focused on EGFR variants conferring sensitivity towards TKI treatments. Recently Tan et al., identified a synonymous variant *rs10251977* (c.2361G>A) present in exon 20 of *EGFR* having greater prognostic implication towards TKI treatments particularly in squamous cell carcinoma of head and neck^7^. In this study, the prevalence of this polymorphism was observed to be 40% (72/180) of GG genotype, 47.2% (85/180) of GA genotype and 12.8% (23/180) of AA genotype among oral cancer patients with a minor allele frequency of 0.36. This is consistent with the previous report where they reported an increased prevalence of this polymorphism in head and neck cancer patients from South Indian origin with 61.24% and 13.95% of both GA and AA genotypes respectively^6^. The study also reported that the polymorphism had a significant risk association in cancer development. However, we did not observe any significant association of this polymorphism with cancer risk. Several studies emphasized the prognostic significance of this EGFR variant in TKI response^22–24^, whereas the wild type genotype predicted to have a good prognosis in metastatic colorectal cancer^25^. Interestingly, Koh et al., reported that this polymorphism is more prevalent in squamous cell carcinoma compared to that of adenocarcinoma of lung, and showed a significant response for gefitinib treatment in patients who carried a wild type allele^26^. Previous study on this variant in advanced oesophageal squamous cell carcinoma recorded that patients with heterozygous genotype had a poor treatment response^27^. The increased prevalence of this variant and its strong association with therapeutic response prompted us to explore its functional significance. The variant allele created a new binding site for miR-891b reducing the level of EGFR-AS1 and leading to increase in the EGFR D/A isoform ratio. Using the online tool lncRNAtor and STITCH database, we have found that lncRNA EGFR-AS1 has a positive interaction with PTBP1 and hnRNPs, major players in alternative splicing and essential for the generation of EGFR-A isoform with both transmembrane and tyrosine kinase domain.

Since the last decade, non-coding RNAs have been one of the main focuses in the field of functional genomics. Emerging evidence has shown the altered expression signatures of a large number of lncRNAs in several human malignancies^28^. In recent years, many of lncRNAs have been proposed to have pivotal role in carcinogenesis. However, the functions and mechanisms of lncRNAs responsible for the development and progression of oral cancer need to be understood. Natural antisense transcripts (NATs) are a group of RNA transcripts that are suggested to play roles in alternative splicing, genomic imprinting, miRNA sponging, also X- chromosome inactivation and they regulate the gene expression both in *cis*- and *trans*- manner^29–31^. *cis*-NAT plays major role in regulation of its sense partner in various manners. A myriad of lncRNA comes under this class of RNA and various studies reported their *cis* role. Recently, we reported a *cis*- mediated regulation of OIP5-AS1 controlling expression of *OIP5* gene by acting as miRNA sponge^32^. Various pan cancer analysis showed the role of deregulated NATs in carcinogenesis and acting as both predictive marker as well as drug targets^33^.

EGFR-AS1, a lncRNA transcribed from the antisense strand of EGFR, was found to be overexpressed in several cancers^11,34–37^. Our study observed a significant upregulation of EGFR-AS1 in oral cancer patients suggesting the oncogenic potential of this lncRNA, which is consistent with above findings. When the EGFR-AS1 expression profile was analysed with reference to genotypes we found a decreased expression of EGFR in GG compared to GA + AA genotype, but without any statistical significance. The altered expression pattern of EGFR-AS1 might be due to previously reported non-canonical RNA editing mechanism which maintains the allele specific lncRNA based on germline polymorphism^38^.

In this study, there was a significant change in the EGFR D:A isoform ratio with the low level expression of EGFR-AS1. Alternative splicing of receptor tyrosine kinases (RTKs) has greater clinical implications, where truncated RTKs arise by alternative splicing of exons or activation of intronic polyA sites^39^. Our bioinformatic analysis revealed that premature termination of EGFR transcript results in the production of EGFR-D isoform by recognition of intronic polyA (IPA) sites and activating premature cleavage and polyadenylation. Additionally, our biomining approach discovered the RNA binding proteins such as PTBP1 and hnRNPU, which were known to play an important role in alternative splicing by significantly interacting with EGFR-AS1. The binding sites for these proteins were present in the skipped exons 15a and 15b, which resides between exons 15 and 16 of *EGFR*. The significant positive correlation between PTBP1 and EGFR-A expression in our study and also TCGA dataset suggest that PTBP1 may mediate alternative splicing of EGFR gene. In agreement with this finding, previous studies have shown that PTBP1 overexpression was known to function as splicing silencer of exon 3 of *BIM* gene by binding to its intron 2^40^ and was also known to act as a splicing reprogrammer of *PKM* gene in pancreatic cancer^41^. Additionally, PTBP1 and PTBP2 are larger family of non-conserved cryptic exons repressors^42^.

Previous studies have established that *SNRNPA* (U1snRNP) prevents the formation of truncated RTK isoforms by negatively controlling the use of IPA sites^35^. Surprisingly, STITCH analysis showed that PTBP1 has a mechanical interaction with SNRNPA which indicates the role of PTBP1 in regulating RTKs isoforms. Besides these several studies emphasized the role of chromatin signatures in alternative splicing Luco et al., reported the role of H3K36me3 in recruiting PTBP1 in *FGFR* exon 3b and promotes alternative splicing of exon 3c in mesenchymal cells^43^. Our analysis for chromatin signatures using UCSC genome browsers showed that *EGFR* exon 15a region is enriched with H3K36me3 mark supporting their role in alternative splicing through PTBP1. Earlier study reported that HuR binding proteins promote alternative splicing of NF1 and FAS transcripts by inducing localized histone hyperacetylation^44^. Recent finding identified EGFR-AS1 and HuR interaction suggesting its role in controlling alternative splicing via chromatin modification^32^. These observations collectively suggest the role of EGFR-AS1 in controlling the expression of EGFR-A from suppressing the formation of EGFR-D.

Certain lncRNAs were shown to competitively sponge the miRNAs and protects its protein coding RNA partners from miRNA mediated repression and modulate their stability. This ceRNA hypothesis is one such phenomenon by which NATs may modulate *cis* regulation in controlling the expression of its sense partner^45^. Our biomining suggested that the genetic variant *rs10251977* in EGFR-AS1 regulates the stability of the lncRNA by miRNA mediated degradation. The minor allele generates a new binding site for miR-891b bringing EGFR-AS1 under the miRNA mediated degradation. This is evident from our results that the decreased level of EGFR-AS1 in tumors that expressed high level of miR-891b. Interestingly, we also found that miR-891b binding site was found in 3′UTR of EGFR-A isoform. We also analyzed another miRNA, miR-138-5p whose binding site is present in both EGFR-AS1 and EGFR-A isoform and its high expression level correlated with reduced level of EGFR-AS1 and EGFR-A. This study showed that both miRNAs were downregulated in tumor and could be tumorsuppressors miRNAs. In addition, recent studies have reported miRNA sponging activity of EGFR-AS1^33,46^ and miR-891b mediated gene regulation^47–49^ supporting the CeRNA function of EGFR-AS1.

The above findings suggest that EGFR-AS1, facilitates alternative splicing of EGFR-A isoform through PTBP1. The genetic variant *rs10251977* in EGFR-AS1 creates binding site for miR-891b which may reduce the level of EGFR-AS1 through miRNA sponging and increasing the EGFR-D/A ratio. The expression analysis of EGFR-A and D isoform, EGFR-AS1 and miR-891b in the TCGA HNSCC dataset suggested a similar role of EGFR-AS1 in regulating EGFR isoform. The functional dissection of miRNA binding site created for miR-891b by the genetic variant *rs10251977* in EGFR-AS1 is warranted. These findings provide an insight on importance of genetic variant *rs10251977* in EGFR-AS1 and the interactions between non-coding RNAs and coding RNA in maintaining cellular homeostasis. Another limitation is our study samples were tumor biopsies which were not enough to carry out additional protein based experiments to confirm the research findings.

## Methods and materials

### Clinical specimens

The present study was conducted after the approval by the Institutional Ethics Committees (IEC) of Madras Medical College, Chennai (No.04092010) and Government Arignar Anna Memorial Cancer Hospital, Kancheepuram (No.101041/e1/2009-2) and also within the ethical framework of Dr. ALM PG Institute of Basic Medical Sciences, Chennai. The blood and tissue samples were collected from Madras Medical College, Chennai and Government Arignar Anna Memorial Cancer Hospital, Kancheepuram during 2009–2014. The study includes a total of 180 participants diagnosed with OSCC and 184 healthy controls. Blood samples were collected to study the genetic variant in *EGFR*. Forty-eight tumor tissue samples and eight normal tissues from individuals free from cancer were collected for expression studies. The patient’s contextual and clinicopathological characteristics were documented with standard questionnaire following the IEC guidelines and written informed consent was obtained from each patient, after explaining about the research study. The tissues were collected in RNAlater solution (Ambion, USA) and transported to the laboratory in cold-storage and stored at – 80 °C.

### DNA isolation

The blood samples were subjected for DNA isolation using Proteinase K digestion and PCI extraction method. The isolated DNA was quantified using NanoDrop2000 UV–Vis spectrophotometer (Thermo Scientific, USA) and its integrity was assessed by resolving in 1% agarose gel in Mupid gel electrophoresis (TaKaRa, Japan) and diluted to 100 ng/μL stored at − 20 °C which was later used for screening genetic variants.

### PCR amplification and sequencing of genetic variant *rs10251977* in EGFR

The exon 20 of *EGFR* gene harbouring the SNP was amplified from 364 DNA samples (180 oral cancer patients and 184 healthy controls) using the sequence specific primers EGFR Fwd:5′- M13- CCTTCTGGCCACCATGCGAA-3′ and Rev:5′-CGCATGTGAGGATCC TGGCT -3′ (where M13 in the EGFR Fwd is the universal sequencing primer 5′-tgtaaaacgacggccagt-3′). Polymerase chain reaction (PCR) was carried in 30 μL volume containing 100 ng of genomic DNA, 1X PCR buffer, 1.5 mM MgCl_2_, 100 μM dNTP (Takara, Japan), 80 nM primers (Sigma, India) and 0.5U of Takara Taq polymerase (Takara, Japan). The PCR is performed using the following thermal conditions: 94 °C for 2 min for initial denaturation, followed by 40 cycles of 94 °C for 30 s, 60 °C for 30 s and 72 °C for 30 s, and 7 min of final extension at 72 °C. The PCR products (298 bp) were electrophoresed in 2% agarose gel. The purified PCR products were sequenced at Macrogen Inc, South Korea.

### RNA isolation and quality control

The tumor samples soaked in RNA later solutions and frozen in − 80 °C were thawed on ice, washed twice with 1 × ice cold PBS, homogenized with zirconium beads using MicroSmash MS-100 automated homogenizer (Tomy Digital Biology, Japan). The total RNA was isolated using the RNAeasy mini kit (Qiagen, Germany) following the manufacturer’s protocol. RNA was quantified using NanoDrop2000 UV–Vis spectrophotometer (Thermo Scientific, USA) and the RNA integrity was assessed by resolving in DEPC treated 1% agarose gel in Mupid gel electrophoresis (TaKaRa, Japan).

### cDNA synthesis and quantitative Real-Time PCR

The cDNA conversion was carried out from total RNA using custom designed miRNA seed specific stem-loop primers for miRNAs (Supplementary Table S5) and a random hexamer primer for coding/non-coding RNAs, with 2 μg of RNA for mRNA and lncRNAs and 10 ng of RNA for miRNAs. The RNA samples were pre-incubated at 65 °C for 20 min followed by 55 °C for 90 min, 72 °C for 15 min and final hold at 4 °C. cDNA conversion was performed using a Reverse Transcription kit (Invitrogen; Thermo Fisher Scientific, Inc. USA) and cDNAs were further diluted 25-fold and stored at − 20 °C.

Real-time RT-qPCR was performed in ABI Quantstudio 6 Flex (ABI Lifetechnology, USA). The reactions were performed in 384 well optical plates with 10 μL total volume using 1 μL of cDNAs as template, TaqMan 2X Universal Master mix (No AmpErase UNG; Thermo Fisher Scientific, Inc. USA), specific forward primers (Supplementary Table S6), universal reverse primer 5′‑TCGTATCCAGTGCGTCGAGT‑3′, and fluorescein amidite‑labeled minor groove binder probes 5′‑CAGAGCCACCTGGGCAATTTT‑3′ for miRNAs expression. The EGFR A/D isoforms and EGFR-AS1 expression levels were analyzed using SYBR‑Green master mix (KAPA SYBR FAST qPCR Kits, USA) with the gene specific primers (Supplementary Table S7), by following the thermal cycling conditions: 50 °C for 2 min and 95 °C for 10 min once, followed by 95 °C for 15 s and 60 °C for 1 min for 40 cycles. GAPDH served as an endogenous control for coding and non-coding genes, and RNU44 as an endogenous control for miRNA. NTC reactions were used in all the experiments. All the reactions were carried out in triplicates, mean Ct was used for analysis and the expression level was calculated using 2^−ΔΔCt^ method.

### In silico approaches

To predict the proteins interacting with EGFR-AS1, lncRNAtor^12^ an online database was used and consensus motifs were predicted using the RBPMap^13^ to identify the RNA binding partners and motifs of interest such as PTBP1 in both EGFR-AS1 and skipped exons of *EGFR*. The motifs with highest stringency were selected for the study. The STITCH online tool was used to collect the molecular partners for PTBP1. UCSC genome browser was used to visualize the epigenetic signal that promotes alternative splicing and also polyA sites to predict premature termination. To assess the functional impact of the polymorphism *rs10251977* in EGFR-AS1, lncRNASNP2 tool was used to identify the loss or creation of binding site for miRNA in the lncRNA or the structural impact on lncRNA was analysed. Gene targets of the miRNA were identified by Targetscan and MiRwalk online tools. Gene Set Enrichment Analysis (GSEA) done by MiRwalk web interface to understand the molecular pathways affected by the selected miRNAs. To confirm the analysis in extended datasets we used three different databases to collect the HNSCC TCGA datasets such as TSVdb^15^, TANRIC^16^ and OMCD^17^ to retrieve the expression status of EGFR isoforms & PTBP1, EGFR-AS1, and miR-891b respectively.

### Statistical analysis

The frequency of alleles and genotypes were compared between the patient’s and control groups by chi-square test. Fisher exact test and odds ratio (OR) with 95% confidence interval (CI) was calculated to find the risk association. Spearman rank correlation analysis and differences between the means were presented as mean ± SEM and analysed using Student’s *t*-test (Mann–Whitney) using Graph Pad Prism statistical software, v 6.01 (Graph Pad software Inc, USA). All tests were two-tailed and a *p* value of < 0.05 was considered as statistically significant.

## Supplementary Information

Supplementary Information 1.

Supplementary Information 2.

Supplementary Information 3.

Supplementary Information 4.
