# Supplementary material for: Genetic variant rs10251977 (G>A) in EGFR-AS1 modulates the expression of EGFR isoforms A and D
Source: Sci Rep. 2021 Apr 22;11:8808. doi: 10.1038/s41598-021-88161-3 (PMC8062556; doi:10.1038/s41598-021-88161-3)
Supplement: Supplementary file 1 — Supplementary Information 1. [file 41598_2021_88161_MOESM1_ESM.pdf]

Supplementary materials

Genetic variant rs*10251977 (G>A)* in EGFR-AS1 modulates the expression of EGFR isoforms A and D

Shankar Dhamodharan[1,+], Mathew Maria Rose[1,+], Reddy Chakkarappan Sundaram[1,4], Karuppiah Vijayamuthuramalingam Uma Dharshini[1], Ramalingam Arulmurugan[2], Shanmugam Subbiah[2], Ituro Inoue[3], Arasambattu Kannan Munirajan[1,*].

[1]Department of Genetics, Dr. ALM PG Institute of Basic Medical Sciences, University of Madras, Taramani Campus, Chennai-600 113, India.

[2]Center for Oncology, Royapettah Government Hospital & Kilpauk Medical College, Royapettah, Chennai-600 014, India.

[3]Division of Human genetics, National Institute of Genetics, Mishima- 411-8540, Japan

[4]Department of Health Research – Multi Disciplinary Research Unit (DHR-MRU), Dr. ALM PG Institute of Basic Medical Sciences, University of Madras, Taramani Campus, Chennai-600 113, India.

[+] These authors contributed equally to this work

[*]**Correspondence to:**

Arasambattu Kannan Munirajan, PhD

Department of Genetics

Dr. ALM PG Institute of Basic Medical Sciences

University of Madras, Taramani Campus

Chennai – 600113, India.

Phone: +91-44-24547064, Fax: +91-44-24540709

E-mail: akmunirajan@gmail.com; akmunirajan@unom.ac.in.

Figure S1

a

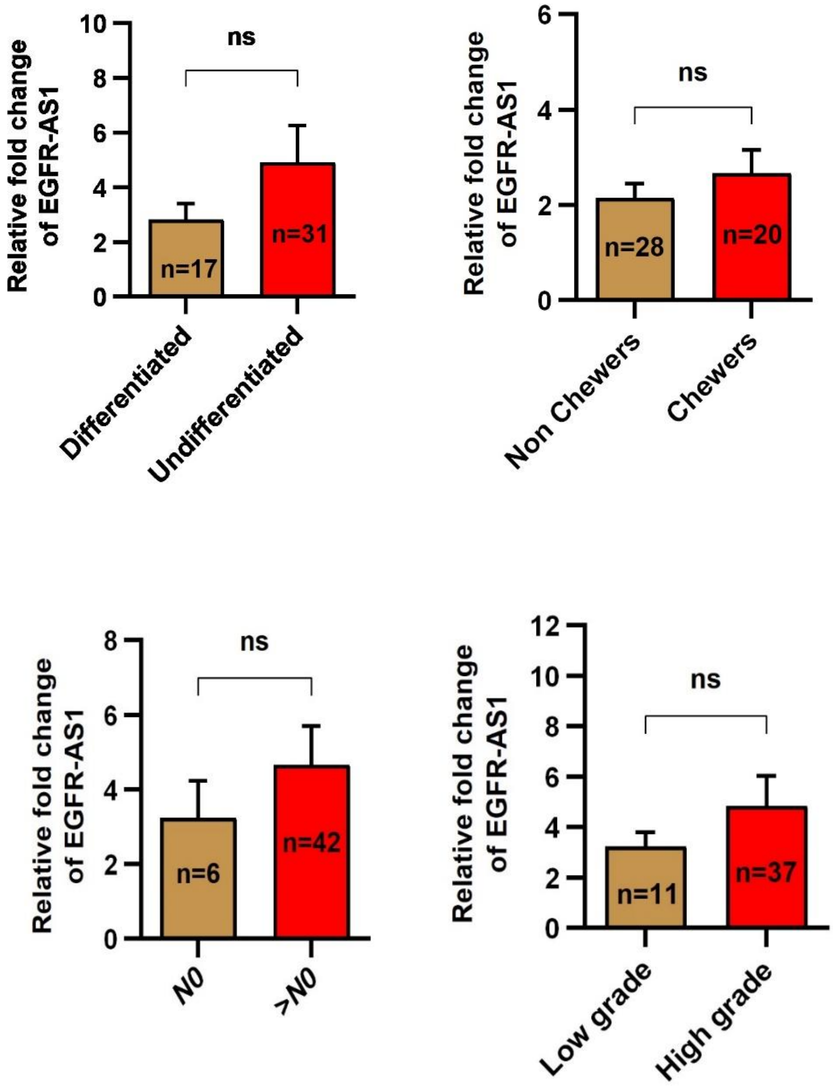
ns
Relative fold change
of EGFR-AS1
n=17
n=31
Differentiated
Undifferentiated
n=28
n=20
Non Chewers
Chewers
n=6
n=42
N0
>N0
n=11
n=37
Low grade
High grade

b

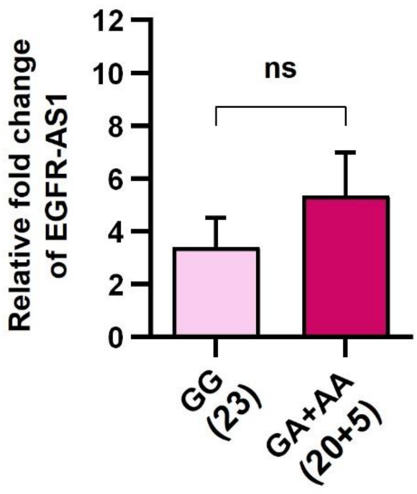
ns
Relative fold change
of EGFR-AS1
GG
(23)
GA+AA
(20+5)

Figure S2

a

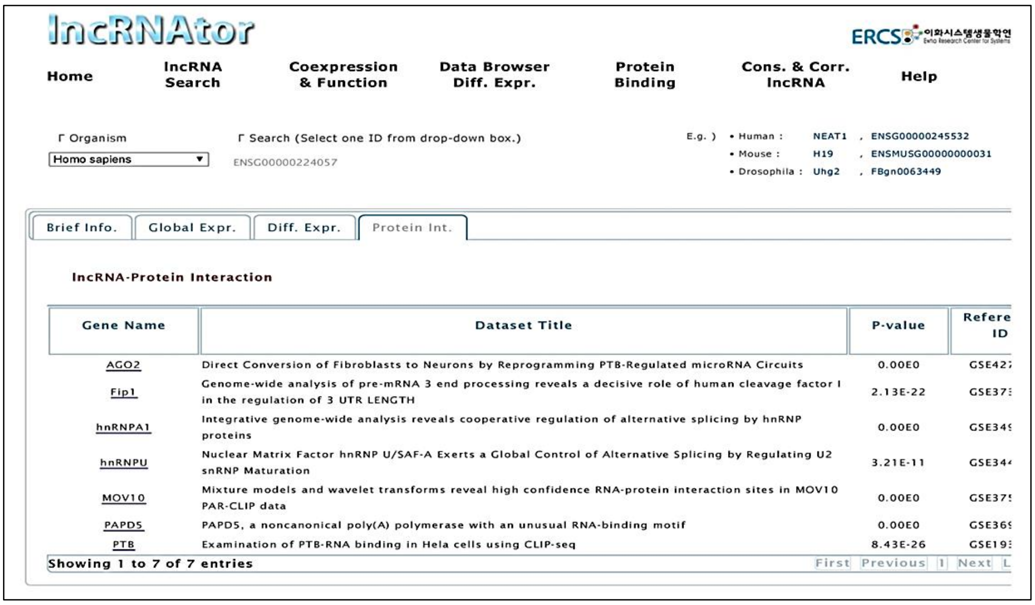

b

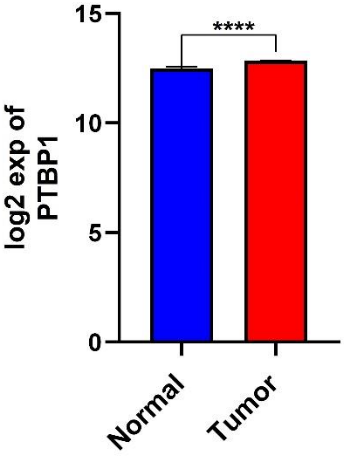

c

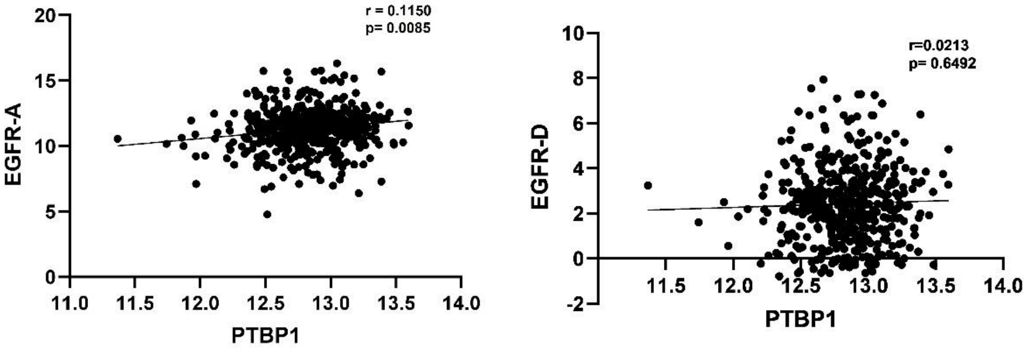

d

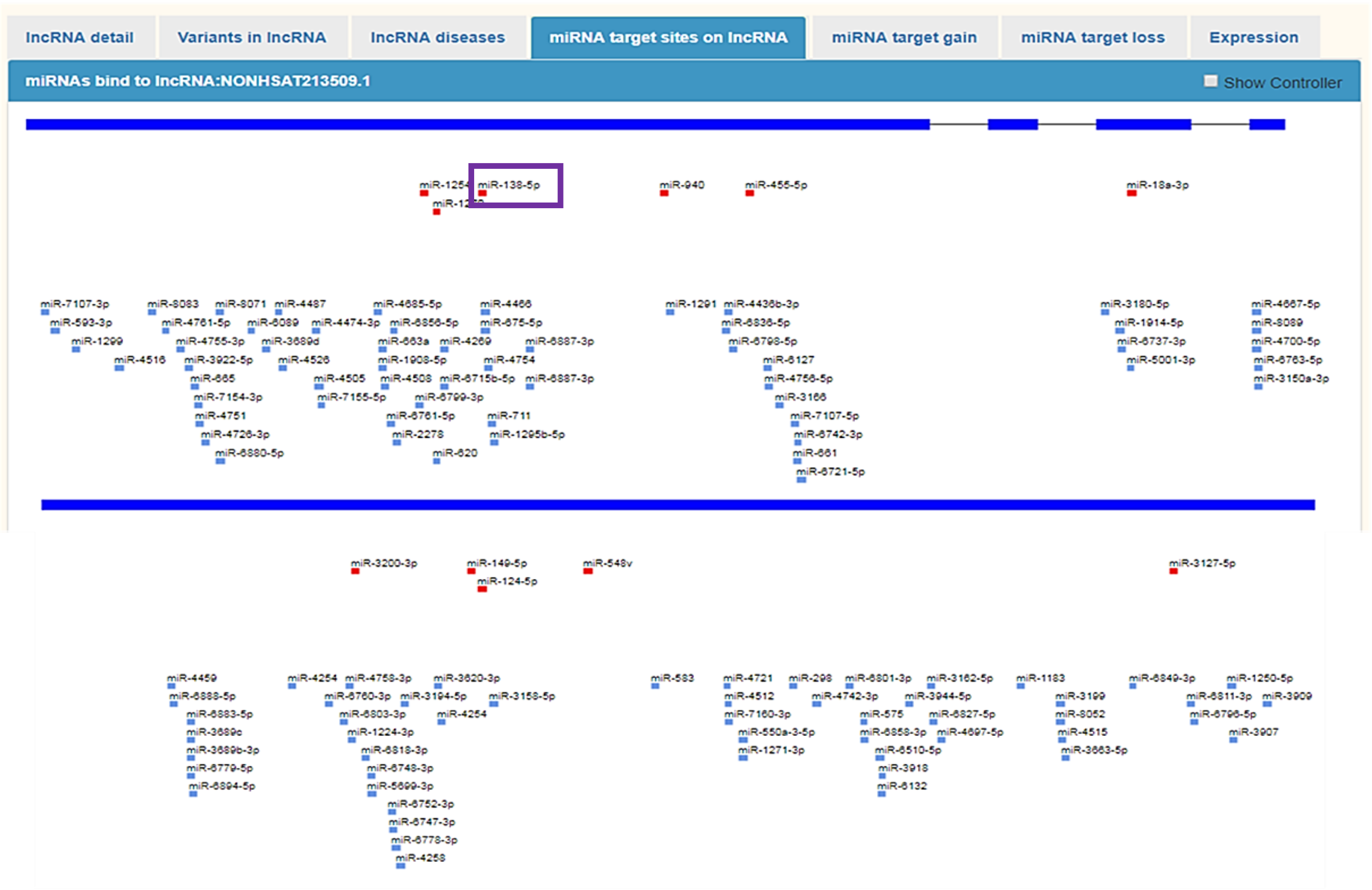

Figure S3

a

b

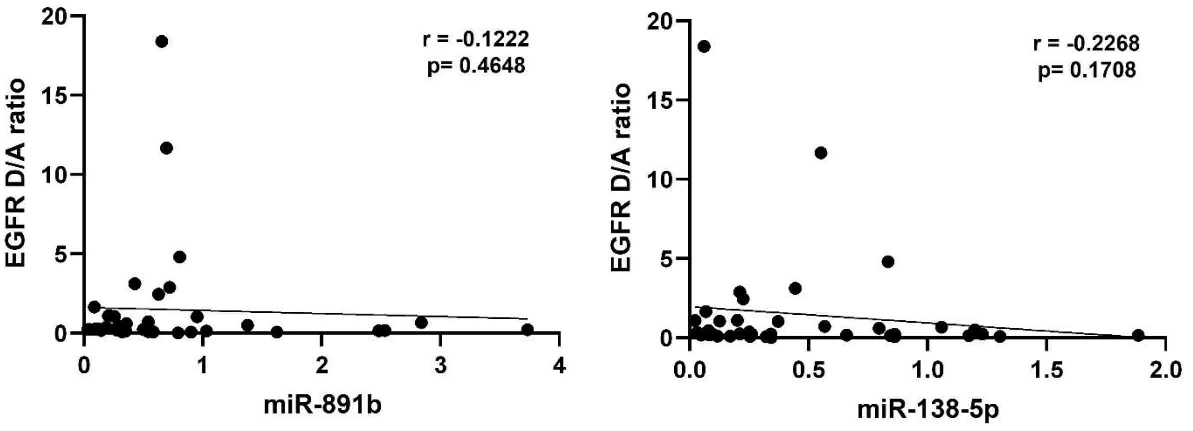

Figure S4

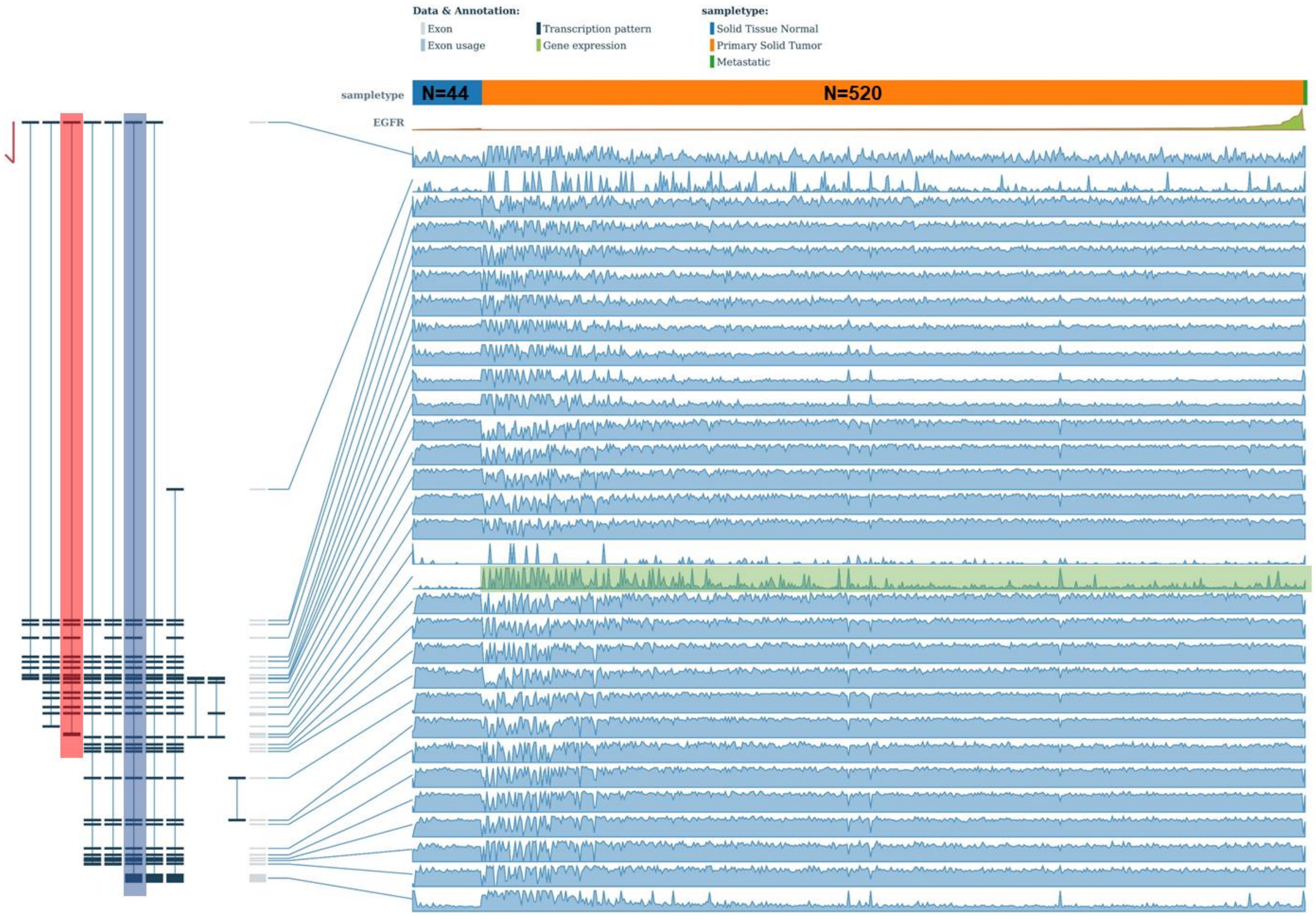

Data & Annotation:
Exon
Exon usage
Transcription pattern
Gene expression
sampletype:
Solid Tissue Normal
Primary Solid Tumor
Metastatic
sampletype
N=44
N=520
EGFR

Table S1. Table showing the prevalence of binding motif of PTBP1 in EGFR-AS1 using RBPmap online tool:

Protein: PTBP1(Hs/Mm)				
Sequence Position	Motif	K-mer	Z-score	P-value
38	ucuu	ucuu	2.58	4.94E-03
213	cucucu	gugucu	2.196	1.40E-02
220	cucucu	cucccu	2.946	1.61E-03
222	cucucu	cccucc	2.25	1.22E-02
224	cucucu	cucccu	2.955	1.56E-03
226	cucucu	cccucu	2.955	1.56E-03
228	cucucu	cucugg	2.259	1.19E-02
233	cucucu	gucccu	2.259	1.19E-02
244	cucucu	aucucu	2.955	1.56E-03
246	cucucu	cucugg	2.205	1.37E-02
253	cucucu	cccccu	1.911	2.80E-02
265	cucucu	cugucg	1.839	3.30E-02
450	ucuu	ucuu	2.161	1.53E-02
451	cucucu	cuuucu	1.92	2.74E-02
481	cucucu	cuggcu	1.839	3.30E-02
492	cucucu	uucugu	1.839	3.30E-02
500	cucucu	cugucu	2.259	1.19E-02
502	cucucu	gucucc	1.884	2.98E-02
523	cucucu	cuaccu	2.625	4.33E-03
532	cucucu	cacucu	3.232	6.15E-04
534	cucucu	cucugc	2.58	4.94E-03
537	cucucu	ugcucu	2.58	4.94E-03
539	cucucu	cucucu	3.607	1.55E-04
541	cucucu	cucuca	3.232	6.15E-04
551	cucucu	cgcccu	2.58	4.94E-03
555	cucucu	cuggcu	2.58	4.94E-03
557	cucucu	ggcucu	2.58	4.94E-03
559	cucucu	cucugg	2.482	6.53E-03
626	cucucu	cgcccu	2.071	1.92E-02
628	cucucu	cccucu	2.839	2.26E-03
630	cucucu	cucugu	2.839	2.26E-03
632	cucucu	cuguca	2.188	1.43E-02
639	cucucu	cuccuu	2.188	1.43E-02
652	cucucu	cucacu	2.813	2.45E-03
1053	cucucu	cuccuu	1.929	2.69E-02
1057	cucucu	uuaucu	1.929	2.69E-02
1059	cucucu	aucucc	1.929	2.69E-02
1061	cucucu	cucccc	1.929	2.69E-02
1064	cucucu	cccucc	1.929	2.69E-02
1066	cucucu	cucccc	1.929	2.69E-02
1072	cucucu	guaucu	1.929	2.69E-02
1074	cucucu	aucucc	1.929	2.69E-02
1076	cucucu	cucccu	2.304	1.06E-02
1081	cucucu	uucccu	1.929	2.69E-02

1098	cucucu	cgaucu	1.839	3.30E-02
1137	ucuu	uauu	1.884	2.98E-02
1142	ucuu	ucuu	2.446	7.22E-03
1148	ucuu	uguu	1.884	2.98E-02
1373	ucuu	ucuu	2.161	1.53E-02
1580	cucucu	aucucu	2.179	1.47E-02
1581	ucuu	ucuc	1.884	2.98E-02
1582	cucucu	cucuga	1.866	3.10E-02
1583	ucuu	ucug	1.884	2.98E-02
1603	cucucu	guaucu	1.884	2.98E-02
1605	cucucu	aucuuu	1.884	2.98E-02
1606	ucuu	ucuu	2.58	4.94E-03
1892	cucucu	cucugc	1.83	3.36E-02
1914	cucucu	agcucu	1.884	2.98E-02
1916	cucucu	cucuuu	2.205	1.37E-02
1917	ucuu	ucuu	2.446	7.22E-03
1918	cucucu	cuuuca	1.732	4.16E-02
1940	ucuu	uuuu	1.884	2.98E-02
2002	cucucu	auuucu	2.563	5.19E-03
2019	cucucu	gucucu	3.277	5.25E-04
2021	cucucu	cucucu	3.679	1.17E-04
2023	cucucu	cucugu	3.304	4.77E-04
2043	cucucu	cucccu	3.277	5.25E-04
2045	cucucu	cccuau	2.491	6.37E-03
2149	ucuu	acuu	2.161	1.53E-02

Table S2. Table showing the prevalence of binding motif of PTBP1 in EGFR 15 and 16 exons using RBPmap online tool:

**Protein: PTBP1(Hs/Mm)**					
**Sequence Position**	**Genomic Coordinate**	**Motif**	**K-mer**	**Z-score**	**P-value**
32	chr7:55165469	cucucu	uccucu	2.045	2.04E-02
34	chr7:55165471	cucucu	cucucu	2.795	2.60E-03
35	chr7:55165472	ucuu	ucuc	1.884	2.98E-02
36	chr7:55165473	cucucu	cucuug	2.179	1.47E-02
37	chr7:55165474	ucuu	ucuu	2.446	7.22E-03
293	chr7:55165730	cucucu	cagucu	2.071	1.92E-02
295	chr7:55165732	cucucu	gucucc	2.071	1.92E-02
313	chr7:55165750	cucucu	guuucu	2.089	1.84E-02
315	chr7:55165752	cucucu	uucucu	2.652	4.00E-03
317	chr7:55165754	cucucu	cucucc	2.652	4.00E-03
325	chr7:55165762	cucucu	auuucu	2.018	2.18E-02
704	chr7:55166141	cucucu	aacucu	1.679	4.66E-02
706	chr7:55166143	cucucu	cucugu	1.982	2.37E-02
764	chr7:55166201	cucucu	cacucu	2.116	1.72E-02
766	chr7:55166203	cucucu	cucuga	1.741	4.08E-02
767	chr7:55166204	ucuu	ucug	1.884	2.98E-02
778	chr7:55166215	cucucu	guaucu	1.741	4.08E-02

781	chr7:55166218	ucuu	ucuu	2.446	7.22E-03
784	chr7:55166221	ucuu	uauu	1.884	2.98E-02
898	chr7:55166335	ucuu	gcuu	2.161	1.53E-02
899	chr7:55166336	cucucu	cuuugu	1.929	2.69E-02
902	chr7:55166339	ucuu	uguu	2.161	1.53E-02
912	chr7:55166349	ucuu	uguu	2.161	1.53E-02
917	chr7:55166354	cucucu	cucauu	2.116	1.72E-02
918	chr7:55166355	ucuu	ucau	2.161	1.53E-02
919	chr7:55166356	cucucu	cauucu	2.116	1.72E-02
921	chr7:55166358	cucucu	uucuuu	2.116	1.72E-02
922	chr7:55166359	ucuu	ucuu	2.714	3.32E-03
923	chr7:55166360	cucucu	cuuucu	2.679	3.69E-03
925	chr7:55166362	cucucu	uucucu	2.67	3.79E-03
926	chr7:55166363	ucuu	ucuc	2.152	1.57E-02
927	chr7:55166364	cucucu	cucuga	2.116	1.72E-02
928	chr7:55166365	ucuu	ucug	2.134	1.64E-02
935	chr7:55166372	cucucu	ccaucu	2.116	1.72E-02
937	chr7:55166374	cucucu	aucugu	2.116	1.72E-02
938	chr7:55166375	ucuu	ucug	2.134	1.64E-02
939	chr7:55166376	cucucu	cuguau	2.116	1.72E-02
956	chr7:55166393	ucuu	ccuu	2.134	1.64E-02
969	chr7:55166406	ucuu	ucuu	3.268	5.42E-04
974	chr7:55166411	ucuu	ccuu	2.438	7.38E-03
976	chr7:55166413	cucucu	uucucu	2.955	1.56E-03
977	chr7:55166414	ucuu	ucuc	2.438	7.38E-03
978	chr7:55166415	cucucu	cucugu	2.955	1.56E-03
979	chr7:55166416	ucuu	ucug	2.438	7.38E-03
986	chr7:55166423	cucucu	cucacu	2.955	1.56E-03
988	chr7:55166425	cucucu	cacuuu	2.25	1.22E-02
989	chr7:55166426	ucuu	acuu	2.438	7.38E-03
990	chr7:55166427	cucucu	cuuucu	2.955	1.56E-03
992	chr7:55166429	cucucu	uucuuu	2.25	1.22E-02
993	chr7:55166430	ucuu	ucuu	3.277	5.25E-04
995	chr7:55166432	cucucu	uuuucu	2.25	1.22E-02
997	chr7:55166434	cucucu	uucugu	2.25	1.22E-02
998	chr7:55166435	ucuu	ucug	2.152	1.57E-02
2334	chr7:55167771	cucucu	cuuucu	1.92	2.74E-02
2350	chr7:55167787	ucuu	ucau	2.098	1.80E-02
2357	chr7:55167794	cucucu	aucucc	1.884	2.98E-02
2358	chr7:55167795	ucuu	ucuc	2.429	7.57E-03
2373	chr7:55167810	ucuu	ucuu	3.214	6.55E-04
2381	chr7:55167818	ucuu	ucuu	3.25	5.77E-04
2383	chr7:55167820	ucuu	uuuu	2.411	7.95E-03
2401	chr7:55167838	ucuu	ucau	2.027	2.13E-02
2404	chr7:55167841	ucuu	uauu	2.027	2.13E-02
2623	chr7:55168060	ucuu	uauu	2.027	2.13E-02

2648	chr7:55168085	ucuu	ucuu	2.688	3.59E-03
2651	chr7:55168088	ucuu	ucau	2.098	1.80E-02
2654	chr7:55168091	ucuu	uauu	2.098	1.80E-02
2656	chr7:55168093	ucuu	uuuu	2.098	1.80E-02
2662	chr7:55168099	ucuu	ccuu	2.134	1.64E-02
2684	chr7:55168121	cucucu	agcucu	1.884	2.98E-02
2686	chr7:55168123	cucucu	cucugu	2.259	1.19E-02
2696	chr7:55168133	cucucu	cacuuu	1.875	3.04E-02
2698	chr7:55168135	cucucu	cuuugu	1.875	3.04E-02
2707	chr7:55168144	cucucu	cucgau	1.884	2.98E-02
2806	chr7:55168243	ucuu	ucug	2.134	1.64E-02
2821	chr7:55168258	ucuu	uguu	2.58	4.94E-03
2823	chr7:55168260	ucuu	uuuu	2.58	4.94E-03
2825	chr7:55168262	cucucu	uuaucu	1.929	2.69E-02
2828	chr7:55168265	ucuu	ucuu	3.554	1.90E-04
2832	chr7:55168269	cucucu	cccccu	1.92	2.74E-02
2834	chr7:55168271	cucucu	cccuuu	1.911	2.80E-02
2835	chr7:55168272	ucuu	ccuu	2.571	5.07E-03
2837	chr7:55168274	ucuu	uuuu	2.571	5.07E-03
2839	chr7:55168276	cucucu	uucuuu	1.911	2.80E-02
2840	chr7:55168277	ucuu	ucuu	3.554	1.90E-04
2841	chr7:55168278	cucucu	cuuucu	2.277	1.14E-02
2844	chr7:55168281	ucuu	ucuu	3.554	1.90E-04
2847	chr7:55168284	ucuu	ucug	2.58	4.94E-03
2867	chr7:55168304	cucucu	guaucu	2.196	1.40E-02
2869	chr7:55168306	cucucu	aucucu	2.848	2.20E-03
2871	chr7:55168308	cucucu	cucugc	2.205	1.37E-02
2881	chr7:55168318	cucucu	auaucu	2.277	1.14E-02
2883	chr7:55168320	cucucu	aucucu	3	1.35E-03
2885	chr7:55168322	cucucu	cucuau	3	1.35E-03
2887	chr7:55168324	cucucu	cuauau	2.277	1.14E-02
2895	chr7:55168332	cucucu	guaucu	2.25	1.22E-02
2897	chr7:55168334	cucucu	aucucu	2.946	1.61E-03
2899	chr7:55168336	cucucu	cucugu	2.946	1.61E-03
2901	chr7:55168338	cucucu	cugugu	2.25	1.22E-02
3044	chr7:55168481	ucuu	uguu	2.134	1.64E-02
3053	chr7:55168490	ucuu	ucau	2.161	1.53E-02
3056	chr7:55168493	ucuu	uuuu	2.161	1.53E-02
3057	chr7:55168494	ucuu	uuuu	2.161	1.53E-02
3060	chr7:55168497	ucuu	ucua	2.161	1.53E-02
3064	chr7:55168501	cucucu	augucu	1.911	2.80E-02
3066	chr7:55168503	cucucu	gucuuu	1.911	2.80E-02
3067	chr7:55168504	ucuu	ucuu	2.723	3.23E-03
3068	chr7:55168505	cucucu	cuuucu	2.277	1.14E-02
3070	chr7:55168507	cucucu	uucuau	1.911	2.80E-02
3071	chr7:55168508	ucuu	ucua	2.161	1.53E-02

3072	chr7:55168509	cucucu	cuauuu	1.911	2.80E-02
3073	chr7:55168510	ucuu	uauu	2.446	7.22E-03
3075	chr7:55168512	ucuu	uuuu	2.446	7.22E-03
3076	chr7:55168513	ucuu	uuuu	2.446	7.22E-03
3077	chr7:55168514	ucuu	uuuu	2.446	7.22E-03
3087	chr7:55168524	ucuu	uccu	2.446	7.22E-03
3097	chr7:55168534	ucuu	ucuu	2.723	3.23E-03
3099	chr7:55168536	cucucu	uucacu	1.92	2.74E-02
3101	chr7:55168538	cucucu	cacugu	1.92	2.74E-02
3103	chr7:55168540	cucucu	cugucu	2.295	1.09E-02
3106	chr7:55168543	ucuu	ucug	2.098	1.80E-02
3107	chr7:55168544	cucucu	cugacu	1.92	2.74E-02
3110	chr7:55168547	ucuu	acuu	2.098	1.80E-02
3116	chr7:55168553	cucucu	gucucc	2.116	1.72E-02
3117	chr7:55168554	ucuu	ucuc	2.152	1.57E-02
3118	chr7:55168555	cucucu	cuccca	2.116	1.72E-02
3120	chr7:55168557	cucucu	cccacu	2.116	1.72E-02
3139	chr7:55168576	cucucu	cuuucu	2.67	3.79E-03
3154	chr7:55168591	cucucu	auuucu	2.071	1.92E-02
3156	chr7:55168593	cucucu	uucucc	2.071	1.92E-02
3158	chr7:55168595	cucucu	cucccu	2.625	4.33E-03
3160	chr7:55168597	cucucu	cccuuu	2.098	1.80E-02
3208	chr7:55168645	ucuu	uuuu	2.134	1.64E-02
3217	chr7:55168654	ucuu	uauu	2.027	2.13E-02
3219	chr7:55168656	ucuu	uuuu	2.098	1.80E-02
3222	chr7:55168659	ucuu	ucuu	2.652	4.00E-03
3244	chr7:55168681	ucuu	uccu	2.152	1.57E-02
3288	chr7:55168725	ucuu	ucuu	3.214	6.55E-04
3291	chr7:55168728	ucuu	uguu	2.375	8.77E-03
3294	chr7:55168731	ucuu	uauu	2.375	8.77E-03
3308	chr7:55168745	ucuu	ucuu	3.214	6.55E-04
3311	chr7:55168748	ucuu	ucuc	2.375	8.77E-03
3399	chr7:55168836	ucuu	uuuu	2.152	1.57E-02
3400	chr7:55168837	ucuu	uuuu	2.152	1.57E-02
3401	chr7:55168838	ucuu	uuuu	2.152	1.57E-02
3402	chr7:55168839	ucuu	uuuu	2.152	1.57E-02
3410	chr7:55168847	ucuu	ucuu	2.688	3.59E-03
3422	chr7:55168859	ucuu	uauu	2.152	1.57E-02
3440	chr7:55168877	cucucu	cauucc	1.83	3.36E-02
3441	chr7:55168878	cucucu	auuccu	1.83	3.36E-02
3445	chr7:55168882	cucucu	cugaau	1.83	3.36E-02
3449	chr7:55168886	cucucu	auauau	1.83	3.36E-02
3454	chr7:55168891	cucucu	uugucu	2.571	5.07E-03
3456	chr7:55168893	cucucu	gucucu	3.223	6.34E-04
3458	chr7:55168895	cucucu	cucucu	3.589	1.66E-04
3460	chr7:55168897	cucucu	cucuca	3.223	6.34E-04

3462	chr7:55168899	cucucu	cucauu	2.58	4.94E-03
3465	chr7:55168902	cucucu	auuucu	2.58	4.94E-03
3467	chr7:55168904	cucucu	uucuca	2.58	4.94E-03
3469	chr7:55168906	cucucu	cucaga	1.839	3.30E-02
3479	chr7:55168916	cucucu	auguau	1.839	3.30E-02
3503	chr7:55168940	ucuu	uguu	2.161	1.53E-02
3515	chr7:55168952	ucuu	acuu	2.152	1.57E-02
3528	chr7:55168965	ucuu	ucuu	2.58	4.94E-03
3537	chr7:55168974	ucuu	acuu	1.884	2.98E-02
3661	chr7:55169098	ucuu	uauu	2.161	1.53E-02
3665	chr7:55169102	ucuu	ucuu	2.723	3.23E-03
3667	chr7:55169104	ucuu	uuuu	2.446	7.22E-03
3668	chr7:55169105	ucuu	uuuu	2.446	7.22E-03
3669	chr7:55169106	ucuu	uuuu	2.446	7.22E-03
3670	chr7:55169107	ucuu	uuuu	2.446	7.22E-03
3671	chr7:55169108	ucuu	uuuu	2.446	7.22E-03
3672	chr7:55169109	ucuu	uuuu	2.446	7.22E-03
3673	chr7:55169110	ucuu	uuuu	2.446	7.22E-03
3674	chr7:55169111	ucuu	uuuu	2.446	7.22E-03
3675	chr7:55169112	ucuu	uuuu	2.446	7.22E-03
3691	chr7:55169128	ucuu	ucuu	2.723	3.23E-03
3694	chr7:55169131	ucuu	uguu	2.161	1.53E-02
3697	chr7:55169134	ucuu	ucug	2.152	1.57E-02
3735	chr7:55169172	cucucu	cucacu	1.929	2.69E-02
3752	chr7:55169189	cucucu	cuccca	1.741	4.08E-02
3865	chr7:55169302	ucuu	uguu	2.134	1.64E-02
3875	chr7:55169312	ucuu	gcuu	2.098	1.80E-02
3880	chr7:55169317	ucuu	ucuu	2.652	4.00E-03
3886	chr7:55169323	ucuu	ucuc	2.134	1.64E-02
3888	chr7:55169325	ucuu	uccu	2.134	1.64E-02
4018	chr7:55169455	ucuu	gcuu	1.884	2.98E-02
4027	chr7:55169464	ucuu	ucuu	2.446	7.22E-03
4033	chr7:55169470	ucuu	uccu	1.884	2.98E-02
4267	chr7:55169704	cucucu	cccucg	2.107	1.76E-02
4269	chr7:55169706	cucucu	cucgcc	2.205	1.37E-02
4273	chr7:55169710	cucucu	ccuucu	2.25	1.22E-02
4281	chr7:55169718	cucucu	cgcucu	2.955	1.56E-03
4283	chr7:55169720	cucucu	cucuca	2.955	1.56E-03
4285	chr7:55169722	cucucu	cucaca	2.25	1.22E-02
4292	chr7:55169729	cucucu	ccuucu	2.25	1.22E-02
4294	chr7:55169731	cucucu	uucucu	2.955	1.56E-03
4296	chr7:55169733	cucucu	cucucc	2.955	1.56E-03
4318	chr7:55169755	cucucu	cacugu	2.188	1.43E-02
4320	chr7:55169757	cucucu	cugucc	2.071	1.92E-02
4323	chr7:55169760	ucuu	uccu	2.152	1.57E-02
4324	chr7:55169761	ucuu	ccuu	2.152	1.57E-02

4333	chr7:55169770	ucuu	gcuu	2.152	1.57E-02
4335	chr7:55169772	cucucu	uucucu	2.259	1.19E-02
4336	chr7:55169773	ucuu	ucuc	2.161	1.53E-02
4337	chr7:55169774	cucucu	cucuuc	1.911	2.80E-02
4338	chr7:55169775	ucuu	ucuu	2.705	3.42E-03
4356	chr7:55169793	ucuu	ucua	2.134	1.64E-02
4357	chr7:55169794	cucucu	cuaccu	1.92	2.74E-02
4360	chr7:55169797	ucuu	ccuu	2.134	1.64E-02
4374	chr7:55169811	ucuu	ucug	2.134	1.64E-02
4383	chr7:55169820	ucuu	gcuu	2.098	1.80E-02
4386	chr7:55169823	ucuu	ucuu	2.58	4.94E-03
4389	chr7:55169826	ucuu	uguu	2.027	2.13E-02
4445	chr7:55169882	ucuu	ucuu	2.446	7.22E-03
4467	chr7:55169904	cucucu	caaucu	2.071	1.92E-02
4470	chr7:55169907	ucuu	ucug	2.027	2.13E-02
4474	chr7:55169911	cucucu	cccucc	2.071	1.92E-02
4476	chr7:55169913	cucucu	cucccu	2.625	4.33E-03
4478	chr7:55169915	cucucu	cccucu	2.625	4.33E-03
4480	chr7:55169917	cucucu	cucugc	2.071	1.92E-02
4537	chr7:55169974	cucucu	gucugu	2.071	1.92E-02
4556	chr7:55169993	cucucu	cuuucu	2.67	3.79E-03
4558	chr7:55169995	cucucu	uucugu	2.116	1.72E-02
4560	chr7:55169997	cucucu	cugucu	2.67	3.79E-03
4562	chr7:55169999	cucucu	gucuca	2.116	1.72E-02
4570	chr7:55170007	cucucu	cuguuu	2.107	1.76E-02
4577	chr7:55170014	cucucu	gugucu	2.107	1.76E-02
4579	chr7:55170016	cucucu	gucugu	2.107	1.76E-02
4821	chr7:55170258	cucucu	cucccc	1.696	4.49E-02
4829	chr7:55170266	cucucu	gccucu	1.804	3.56E-02
4831	chr7:55170268	cucucu	cucuca	2.241	1.25E-02
4833	chr7:55170270	cucucu	cucaca	1.902	2.86E-02
4839	chr7:55170276	ucuu	uauu	2.098	1.80E-02
4850	chr7:55170287	ucuu	acuu	2.098	1.80E-02
4856	chr7:55170293	cucucu	ccaucu	2.089	1.84E-02
4858	chr7:55170295	cucucu	aucuuu	1.875	3.04E-02
4859	chr7:55170296	ucuu	ucuu	2.58	4.94E-03
4860	chr7:55170297	cucucu	cuuucu	2.25	1.22E-02
4862	chr7:55170299	cucucu	uucucc	1.884	2.98E-02
4863	chr7:55170300	ucuu	ucuc	2.098	1.80E-02

Supplement Table S5. List of Universal reverse transcription primers used for cDNA synthesis:

Study type	Primer type	Sequence
miRNA	Stem loop RT primer – RNU44 specific	5`- GTCGTATCCAGTGCGTCGAGTGACACGAGAGCCA CCTGGGCAATTTGCACTGGATACGAC**AGTCAG**- 3`
	Stem loop RT primer – miR-891b specific	5`- GTCGTATCCAGTGCGTCGAGTGACACGAGAGCCA CCTGGGCAATTTGCACTGGATACGAC**TCAATG** - 3`
	Stem loop RT primer – miR-138-5p specific	5`- GTCGTATCCAGTGCGTCGAGTGACACGAGAGCCA CCTGGGCAATTTGCACTGGATACGAC**CGGCCT** - 3`

Supplement Table S6. List of gene specific forward primers used for real time PCR experiments:

ncRNAs	Forward primer sequences
RNU44	5`- GCAAATGCTGACTGAACATGA - 3`
miR-891b	5`- CAGTGCAACTTACCTGAGTC - 3`
miR-138-5p	5`- AGCTGGTGTTGTGAATCAG - 3`

Supplement Table S7. List of primers used for SYBR® Green gene expression assays:

Gene	Forward	Reverse
GAPDH	5` - AGGGCTGCTTTTAACTCTGGT - 3`	5` - CCCCACTTGATTTTGGAGGGA - 3`
EGFR - A	5` - TGCACAGGACGGGGACCAGAC - 3`	5` - GCTTGGTTGGGAGCTTCTCCACT - 3`
EGFR - D	5` - CCAAACTGCACCTACGGGCCAG - 3`	5` - GAGGCCGTGATGATCACAGATGCAG - 3`
EGFR - AS1	5` - GGAGCGCAGACCGCATGTGAG - 3`	5` - GCCACCATGCGAAGCCACACTG - 3`
PTBP1	5` - CATCACGCTCTCGAAGCACCAG - 3`	5` - GAGATCCTCCTCGGAGACTGAGG - 3`
