# Supplementary material for: Genetic variant rs10251977 (G>A) in EGFR-AS1 modulates the expression of EGFR isoforms A and D
Source: Sci Rep. 2021 Apr 22;11:8808. doi: 10.1038/s41598-021-88161-3 (PMC8062556; doi:10.1038/s41598-021-88161-3)
Supplement: Supplementary file 4 — Supplementary Information 4. [file 41598_2021_88161_MOESM4_ESM.docx]

**Supplementary material**

**Figure S1**

**a.** Graph showing the relative fold change of EGFR-AS1 in relation to clinicopathological characteristics. **b.** Relative fold change of EGFR-AS1 in patients with GG and GA+ AA genotype of *rs10251977*.

**Figure S2**

**a.** Predicted EGFR-AS1 interacting proteins using online database lncRNAtor.

**b**. Relative expression level of PTBP1 in HNSCC TCGA dataset. **c**. Scatter plot showing the correlation analysis of EGFR - A and D isoforms expression in relation to PTBP1 expression.

**d.** LncRNASNP2 showing the miRNA target sites, with miR-138-5p (within box) is targeted independent of the variant *rs10251977*.

**Figure S3**

**a.** Scatter plot showing the correlation analysis of EGFR D/A ratio level in relation to miR-891b levels.

**b.** Scatter plot showing the correlation analysis of EGFR D/A ratio level in relation to miR-138-5p levels.

**Figure S4**

TSVdb TCGA dataset showing the splice variants of EGFR (Red box – EGFR-D isoform, Blue box -EGFR-A isoform, Green box – Exon 15b usage in HNSCC-TCGA tumor samples).

**Table S1 Table showing the prevalence of binding motif of PTBP1 in EGFR-AS1 using RBPmap online tool**

**Table S2 Table showing the prevalence of binding motif of PTBP1 in EGFR 15 and 16 exons using RBPmap online tool**

**Table S3 miR-891b miRWalk_GSEA_results (Excel file)**

**Table S4 miR-138-5p miRWalk_GSEA_results (Excel file)**

**Table S5 List of Universal reverse transcription primers used for cDNA synthesis**

**Table S6 List of gene specific forward primers used for real time PCR experiments**

**Table S7 List of primers used for SYBR Green gene expression assays**
